# Supplementary material for: Complexome profiling of the Chlamydomonas psb28 mutant reveals TEF5 as an early PSII assembly factor
Source: Plant Cell. 2025 Mar 17;37(6):koaf055. doi: 10.1093/plcell/koaf055 (PMC12164590; doi:10.1093/plcell/koaf055)
Supplement: koaf055_Supplementary_Data [file koaf055_supplementary_data.zip › psb28_interactive.zip!/plots/intensity/_Cre01.g016514.t1.1.html]

|  |  |
| --- | --- |
| **Identifier** | Cre01.g016514 |
| **Trivial name** |  |
| **Euclidean distance** | 5266.42 |
| **Average intensity sum (WT)** | 321.4 || **Average intensity sum (Mut)** | 403.3 |
| **Adjusted euclidean distance** | 13.06 |
| **MapMan annotation** | TCA / organic transformation.TCA.pyruvate DH.E3 lipid metabolism.FA synthesis and FA elongation.pyruvate DH |
| **p-value** (Welch test) of intensity sums | 0.37366 |
